# Evaluation of the Effect of Antihypertensive Drugs on the Values of Dental Pulp Oxygen Saturation in Hypertension Patients: A Case-Control Study

**DOI:** 10.7759/cureus.33245

**Published:** 2023-01-02

**Authors:** Wafaa Almosallam, Abeer A Aljoujou, Helen Rushdi Ayoubi, Hasan Alzoubi

**Affiliations:** 1 Department of Oral Medicine and Radiology, Damascus University, Damascus, SYR; 2 Department of Oral Medicine, Damascus University, Damascus, SYR; 3 Department of Endodontic and Operative Dentistry, Damascus University, Damascus, SYR; 4 Department of Pediatric Dentistry, Damascus University, Damascus, SYR

**Keywords:** pulse oximeter, dental pulp oximetry, systemic oximetry, antihypertensive drugs, hypertension

## Abstract

Purpose

This study aimed to know about the positive or negative effect of antihypertensive drugs of different groups on the values of dental pulp oxygen saturation in hypertension patients.

Materials and Methods

A case-control study to evaluate the impact of the antihypertensive drugs on the values of dental pulp oxygen saturation in hypertension patients. The studied sample consisted of 40 participants, and they were distributed into two groups: Group I (n=20): Hypertension patients treated with antihypertensive drugs, and Group II (n=20): Healthy participants. A finger pulse oximeter was recorded after a rest period of 15 minutes by BCI® Advisor® vital signs monitor. The patient was then asked to use a chlorhexidine digluconate mouth rinse for five minutes, and the two dental pulp pulse oximeters for the central upper incisors were also recorded for all participants. Data were analyzed using the Mann-Whitney U test.

Results

​​​​​​The results showed that there was no significant difference between the finger pulse oximeters of the two studied groups (P-value = 0.421). The two dental pulp oxygen saturation was higher than the control group with statistically significant (P-value = 0.043, P-value = 0.002).

Conclusions

Within the limitation of this study, it can be concluded that antihypertensive drugs increase the dental pulp oxygen saturation in patients with hypertension who are treated with antihypertensive drugs, and thus there is a positive effect of these drugs in stimulating the dental pulp.

## Introduction

Hypertension is a cardiovascular disease that causes premature death, with a worldwide prevalence of about 31.1% [1]. Hypertension is classified into essential, which constitutes about 90% of patients, and secondarily associated with systemic diseases and disorders, which constitutes 10% of patients [2].

The management of patients with hypertension is divided into behavioral management based on lifestyle changes such as weight loss, reduced sodium intake, and increased physical activity [3] and pharmacological management with antihypertensive drugs in their different groups: angiotensin-converting enzyme inhibitors (ACEIs), angiotensin II receptor blockers (ARBs), dihydropyridine calcium channel blockers, thiazide, and thiazide-like diuretics, and beta-adrenergic receptor blockers [4].

The dental pulp is a highly vascularized tissue located in a non-stretchable environment surrounded by rigid dentin walls, the apical foramen, and accessory canals are the only entrance to the tooth [5]. The apical foramen, with its terminal branches, forms a vascular network beneath the odontoblast cells; they nourish the tissues, mediate several acute or chronic inflammatory mechanisms, as well as have a restorative function for pulp and dentin tissues [6].

The tests that rely on the passage of light through the tooth are a suitable means of assessing vascular and pulp vitality. A dental pulp oximeter is an effective and objective method of monitoring oxygen saturation [7]. The visual diagnostic techniques for pulp blood flow are associated with patient comfort and ease of application, especially pulse oximetry [8].

The principle of the pulse oximetry technique is based on the spectroscopy technique of hemoglobin as a light-absorbing component within the blood (both oxygenated and deficient). The red oxygenated hemoglobin absorbs more infrared radiation (940 nm); while passing and reflecting the red color, and the dark red-black deficient hemoglobin absorbs more red light (660 nm) while passing and reflecting the blue color [9].

The oximetry system (as well as for the dental pulp) consists of a monitoring screen, a processor, and sensors [10] so that the vascular is confined between two adjacent light sources and a corresponding receiver that receives the remaining red and infrared light that passed through the vascular [11]. The captured optical energy was converted into electricity that transmits the received signal to the microprocessor, where it passes through an amplified circuit more than 100 times and another filter to remove optical noise as much as possible, then the received signals are analyzed, and the results are compared with manufacturer stored values [11].

Therefore, this study was conducted to assess the potential association between antihypertensive drugs and the values of dental pulp pulse oximetry in hypertensive patients.

There is very little literature devoted specifically to the use of pulse oximeters with hypertension patients. Therefore, this study used pulse oximetry to determine the level of oxygen saturation in the pulp of intact maxillary central incisors to evaluate the correlation between antihypertensive drugs of different groups and the level of oxygen saturation in the dental pulp.

## Materials and methods

A case-control study to evaluate potential association between the antihypertensive drugs and the values of dental pulp oxygen saturation in hypertension patients compared with healthy participants. The study protocol was approved by the Scientific Research and Postgraduate Board of Damascus University Ethics Committee of Damascus University, Damascus, Syria (IRB No. UDDS689-1402020/SRC-1450).

The sample size was determined using a sample size calculation program (PS Power and Sample Size Calculation Program, Version 3.0.43). The sample size was calculated using outcomes from Stella et al., comparing oxygen saturation in dental pulp of permanent teeth between children/adolescents [12]. Sample size calculation produced a required sample size of 40 participants to detect a significant difference (90% power, two-sided 5% significance level). The studied sample was divided equally into two groups: Group I (n=20): Hypertension patients treated with antihypertensive drugs, and Group II (n=20): Healthy participants.

Inclusion criteria were patients diagnosed with hypertension using oral antihypertensive drugs and at least two maxillary central incisors are intact. While exclusion criteria were smokers, alcoholics, and systemic disease that affects the oral mucosa, such as diabetes mellitus. The sample was collected from patients diagnosed with hypertension attending the cardiology department at Al-Assad University Hospital. Written informed consent to participate in the study was obtained from all patients.

**Figure 1:**
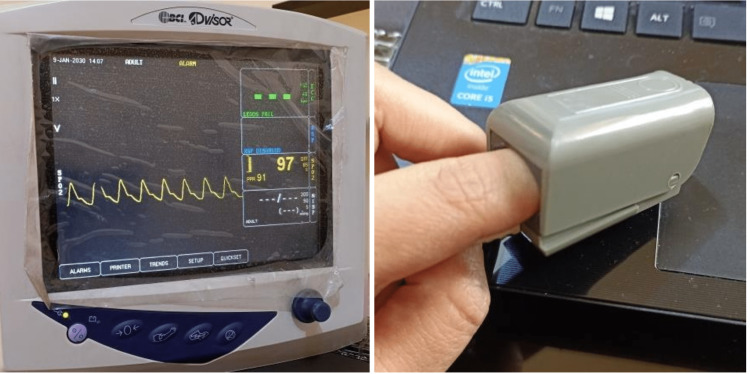
The Monitor used in this study.

A comprehensive oral examination (hard and soft tissue) was performed for all sample members then finger pulse oximetry was carried out for all patients in both groups using a finger probe connected to the Monitor device (BCI® Advisor®, Smiths Medical ASD, Inc., USA) (Figure 1) after a rest period of 15 minutes and in a uniform sitting position for all patients.

**Figure 2:**
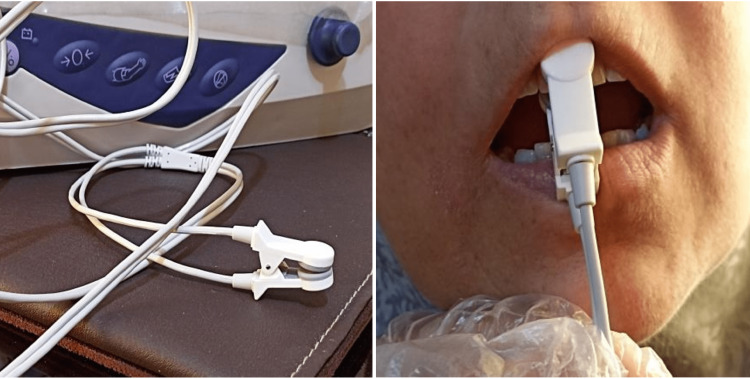
The ear sensor and measurement of the dental pulp oximetry.

Then, the patients were asked to use a 0.12% chlorhexidine digluconate mouth rinse for five minutes to reduce the presence of bacteria, and the central upper incisors were cleaned using a slow-speed handpiece brush and pumice powder to measure the value of oxygen saturation of the dental pulp, using an ear probe (Figure 2).

The two maxillary central incisors were isolated with cotton rolls, and then the light-emitting probe part was placed on the buccal surface of the tooth and the light-receptive part on the palatal surface of the same tooth. Each of the sensor pairs was confined to the middle third of the tooth without touching the soft tissues and avoiding any movement from the patient when taking the readings, and all the mean observed values for the two maxillary central incisors were recorded.

Statistical analysis was performed by using the IBM Corp. Released 2012. IBM SPSS Statistics for Windows, Version 21.0. Armonk, NY: IBM Corp. The data were analyzed with Mann-Whitney U Test. The testing was performed at α=0.05.

## Results

The studied sample consisted of 20 hypertension patients who were treated with antihypertensive drugs, their ages ranged between 44 and 68 years, with an average of 57.10 ± 7.55 (30% males and 70% females), and 20 healthy participants, their ages ranged between 40 and 63 years, with an average of 48.30 ± 6.52 (25% males and 75% females) (Table 1). In the hypertension patients group, beta-blockers were the most used drug at 55%, followed by calcium blockers at 25% (Table 2).

**Table 1:** Basic sample characters

Groups	Gender		Ages			
	Male	Female	Min	Max	mean	SD
Group I	30%	70%	44.00	68.00	57.10	7.55
Group II	25%	75%	40.00	63.00	48.30	6.52

**Table 2:** The type of drugs used.

The type of drugs used					
Beta blockers	ACEIs	ARBs	Calcium channel blockers	ACEIs and calcium channel blockers	ARBs and calcium channel blockers
55.0%	5.0%	0.0%	25.0%	5.0%	10.0%

To study the potential association effect of antihypertensive drugs on the finger and dental pulp pulse oximeter values ​​between both groups, the Mann-Whitney U test was conducted. The results have shown no significant difference in the systemic pulse oximeter values ​​between both groups (P-value = 0.421), while a significant difference was found in the two dental pulp pulse oximeter values (P-value = 0.043, P-value = 0.002) for the first and second measurement, respectively (Table 3).

**Table 3:** Mann-Whitney U test results for the differences between the means of systemic and dental pulp pulse oximeter between groups.

Pulse oximeter measurement	Groups	Mean	Min	Max	Standard deviations	P-value
Finger pulse oximeter	Group I	95.85	93.00	98.00	1.18	0.421
	Group II	95.50	92.00	99.00	1.76	
First dental pulp pulse oximeter	Group I	83.70	73.00	90.00	4.21	0.043
	Group II	77.05	67.00	85.00	4.17	
Second dental pulp pulse oximeter	Group I	84.20	73.00	95.00	6.01	0.002
	Group II	76.90	68.00	84.00	3.14	

## Discussion

The pharmacological treatment of hypertensive patients is represented by various groups of antihypertensive drugs. ACEIs are the first treatment because it dilates blood vessels and lowers blood pressure. This is because this group blocks the conversion of one of the enzymes in the body that produces angiotensin II [13].

ARB's drugs also block angiotensin receptors on the surface of blood vessels, causing veins and arteries to relax, narrowing blood vessels and increasing blood pressure [14]; in addition, thiazide and thiazide-like diuretics reduce blood volume and lead to a direct relaxation of vascular smooth muscle [13]. Beta-blockers mainly vasodilate, while calcium channel blockers bind to calcium channels in the cell membrane, preventing an increase in calcium levels and thus expanding peripheral arteries [15,16].

Thus, all these groups relied mainly on vasodilation, and because the dental pulp is a tissue with a unique vascular and nervous structure and high biological activity [17], this study was conducted to investigate the potential association between the antihypertensive drugs and pulp oximetry measurements using an ear probe. Goho demonstrated the ability to measure pulp oxygen saturation levels in primary and permanent teeth using an ear probe [18].

There was a significant difference between each of the dental pulp pulse oximeter values of the first measurement (P-value = 0.043) and the second measurement (P-value = 0.002) between the groups of hypertensive patients and healthy patients, where the pulse oximeter values in the studied sample were higher than the control sample, and the potential possible explanation for this might be the mechanism that antihypertensive drugs play a role in dilating blood vessels [4]. This dilating is a mechanism to enhance blood flow to areas of the body that are lacking in oxygen and nutrients [19]. Thus, the value of the pulse oximeter of the dental pulp will increase.

While there was not a significant difference between the values of finger pulse oximeter between the two groups (P-value = 0.421), this can be explained by the unique pulp structure, which is characterized by the dental pulp and its confined location within the hard dental tissue and therefore it is more sensitive to the values of oxygenation. The results of this study agreed with Wilson et al. regarding the effect of dental oxygenation values in children with sickle cell anemia and with the systemic oxygenation values that remained the same in the two groups [20].

The explanation for lower oxygen saturation values in teeth compared to the finger can be that the diffraction of infrared light by the enamel prisms and dentin may cause a decrease in oxygen saturation values and light scatter through the gingiva.

Antihypertensive drugs led to activation of the pulp tissue and increased blood supplies without any complaints of pain, as there was no inflammatory or infection process, as oxygen saturation values decrease in inflammatory conditions due to a decrease in blood vessels in the pulp [21]. This is can be explained by the Low-Compliance System theory, which states that the inflammatory process causes vasodilation of the arterioles present in the pulp tissue [22], and since the pulp is surrounded by hard, non-compliant tissues; therefore a rise in the pressure of the interstitial fluid occurs, causing pressure on the walls of blood vessels, which reduces the blood supply loaded with oxygen [23].

The diversity of drug groups and their different effects in the oral cavity, the great need for drug combinations between two or more groups, and our inability to limit the direct effect of each group separately, as well as the long-term treatment of these drugs, are the major limitation of the study.

## Conclusions

Within the limits of this study, there is a rise in the values ​​of dental pulp oximetry in patients with hypertension who are treated with antihypertensive drugs without the presence of any symptoms, and thus there is a potential association between anti-hypertensive drugs and pulp oxygen saturation levels.
